# Supplementary material for: Diversity and Cytogenomic Characterization of Wild Carrots in the Macaronesian Islands
Source: Plants (Basel). 2021 Sep 18;10(9):1954. doi: 10.3390/plants10091954 (PMC8473144; doi:10.3390/plants10091954)
Supplement: Supplementary file 1 [file plants-10-01954-s001.zip › plants-1343093-supplementary.pdf]

plants

MDPI

Supplementary Data

Diversity and cytogenomic characterization of wild carrots in the Macaronesian Islands

Guilherme Roxo, Mónica Moura, Pedro Talhinhas, José Carlos Costa, Luís Silva, Raquel Vasconcelos, Miguel Menezes Sequeira, and Maria Manuel Romeiras

Table S1: Raunkier classification of the sampled taxa

Taxon	Raunkier Classification
Daucus carota L. subsp. *azoricus* Franco	Hemicryptophyte
Daucus carota L. subsp. *carota*	Hemicryptophyte
Daucus carota subsp. *gummifer* (Syme) Hook.f.	Hemicryptophyte
Daucus carota L. subsp. *halophilus* (Brot.) A. Pujadas	Hemicryptophyte
Daucus carota L. subsp. *maximus* (Desf.) Ball	Hemicryptophyte
Daucus carota L. subsp. *sativus* (Hoffm.) Arcang	Hemicryptophyte
Daucus crinitus Desf.	Hemicryptophyte
Daucus muricatus (L.) L.	Therophyte
Melanoselinum decipiens (Schrad. & J.C.Wendl.) Hoffm.	Chamaephyte or Microphanerophyte*
Monizia edulis Lowe	Chamaephyte
Pseudorlaya pumila (L.) Grande	Therophyte
Tornabenea annua Bég.	Hemicryptophyte
Tornabenea bischoffii J.A. Schmidt	Chamaephyte
Tornabenea insularis (Parl. Ex Webb) Parl. Ex Webb	Hemicryptophyte
Tornabenea ribeirensis Schmidt & Lobin	Hemicryptophyte
Tornabenea tenuissima (A. Chev.) A. Hansen & Sunding	Chamaephyte

* On the statistical analysis we followed the classification of Frankiewicz et al. [20].

Table S2: Phytosociological vegetation units of the sampled taxa

Taxa	Phytosociological Vegetation Unit
Daucus carota L. subsp. *azoricus* Franco	*Euphorbio azoricae – Festucion petraeae*
	Tolpido succulentae – Agrostion congestiflorae
	Holco rigidi – Brachypodietum gaditanae
Daucus carota L. subsp. *carota*	*Artemisietea vulgaris*
Daucus carota subsp. *gummifer* (Syme) Hook.f.	*Crithmo – Armerietalia*
Daucus carota L. subsp. *halophilus* (Brot.) A. Pujadas	*Crithmo maritimi – Daucion halophili*
	Scrophulario sublyratae – Lavateretum arboreae
Daucus carota L. subsp. *maximus* (Desf.) Ball	*Onorpodion castellani*
Daucus crinitus Desf.	*Hyparrhenion hirtae*
Daucus muricatus (L.) L.	*Hordeion leporini*
Melanoselinum decipiens (Schrad. & J.C.Wendl.) Hoffm.	*Euphorbion melliferae*
	Isoplexido sceptri – Euphorbietum melliferae
Monizia edulis Lowe	*Sinapidendro angustifolii – Aeonion glutinosi*
	Monizia edulis community
Pseudorlaya pumila (L.) Grande	*Vulpietalia*
Tornabenea annua Bég.	*Globulario amygdalifoliae – Periplocion chevalieri*
	Echio hypertropici – Euphorbietum tuckeyanae
	Dichrostachyo platycarpae – Acacietum caboverdeanae
	Heteropogonetum melanocarpi
Tornabenea bischoffii J.A. Schmidt	*Globulario amygdalifoliae – Periplocion chevalieri*
	Loto latifolii – Artemisietum gogonei
	Melanoselino bischoffii – Globarietum amygdalifoliae
	Dichanthio foveolati – Heteropogonetum contorti

Tornabenea insularis (Parl. ex Webb) Parl. ex Webb	*Globulario amygdalifoliae – Periplocion chevalieri*
	Cocculo penduli – Sarcostemmetea daltonii
	Aeonio gorgonei – Sarcostemmetum daltonii
	Echio stenosiphonis – Euphorbietum tuckeyanae
	Tetraeno waterlotii – Sarcostemmetum daltonii
	Launaeo thalassicae – Euphorbietum tuckeyanae
	Asterisco smithii – Euphorbietum tuckeyanae
	Forsskaoleo procridifoliae – Ficetum gnaphalocarpae
	Campanulo bravensis – Launaetum thalassicae
Tornabenea ribeirensis Schmidt & Lobin	*Dichrostachyo platycarpae – Acacietalia caboverdeanae*
	Fico gnaphalocarpae – Acacion caboverdeanae
Tornabenea tenuissima (A. Chev.) A. Hansen & Sunding	*Globulario amygdalifoliae – Periplocion chevalieri*
	Echietum vulcanori
	Erysimo caboverdeanae – Periplocetum chevalieri

Table S3: Description of the phytosociological vegetation units of the sampled taxa

Phytosociological Vegetation Unit	Description
Aeonio gorgonei – Sarcostemmetum daltonii Rivas-Martínez, Lousã, J.C. Costa & M.C. Duarte 2017	Climactical and edapho-xerophilous dwarf shrub savanna community rich in succulent plants well-developed on S. Nicolau and São Vicente Islands on skeletal leptosols ancient altered volcanic rocky, in tropical desertic, thermo-tropical arid bioclimate, often affected by the trade winds from the north, especially in winter.
Artemisietea vulgaris Lohmeyer, Preising & Tüxen in Tüxen 1950 ex von Rochow 1951	Pioneer and ruderal sunny vegetation composed by perennial and tall biennial forbs, grasses, and thistles. These communities prosper in deep soils enriched in nitrogen (due to man or cattle action). In Mediterranean pluviseasonal and temperate bioclimate. Holarctic distribution, neophytes in all tropical areas.
Asterisco smithii – Euphorbietum tuckeyanae Rivas-Martínez, Lousã, J.C. Costa & M.C. Duarte 2017	Climactical closed shrub savanna community growing on coluvisols and leptic andosols.
Campanulo bravensis – Launaetum thalassicae Rivas-Martínez, Lousã, J.C. Costa & M.C. Duarte 2017	Thermotropical arid to semiarid chasmophytic association, occurs on volcanic rocks of Brava Island
Cocculo penduli – Sarcostemmetea daltonii Rivas-Martínez, Lousã, J.C. Costa & M.C. Duarte 2017	Micro-shrubland often succulent and deciduous micro-woodland open savanna of Cabo Verde, mostly with aridic tropical bioclimate; occurring in desertic, xeric, infra-thermo-meso to lower supratropical ultrahyperarid to upper dry, strongly euhyperoceanic bioclimate; growing on arenosols, andosols, leptosols (lithic, skeletic, hyperskeletic) or fluvi-regosols and sometimes on little altered volcanic materials (lava and tephra).
Crithmo – Armerietalia Géhu & Géhu-Frank 1984	Atlantic chasmophytic aerohaline pioneer communities of sea cliffs.
Crithmo maritimi – Daucion halophili Rivas-Martínez, Lousã, T.E. Díaz, Fernández-González & J.C. Costa 1990	Rupicolous dwarf-herb vegetation of salt-sprayed cliffs of the southwestern Iberian Peninsula and Northern Morocco.
Dichanthio foveolati – Heteropogonetum contorti Rivas-Martínez, Lousã, J.C. Costa & M.C. Duarte 2017	Xeromorphic perennial grassland savanna, growing on leptic andosols, in upper thermotropical to lower mesotropical and from lower semiarid to dry bioclimates.
Dichrostachyo platycarpae – Acacietalia caboverdeanae Rivas-Martínez, Lousã, J.C. Costa & M.C. Duarte 2017	Deciduous micro-woodland climactic savanna (*Fico gnaphalocarpae-Acacion caboverdeanae*), growing on lithosols, arenosols, andosols and tephras, including the edaphohygrophilous coastal palm groves (*Phoenicion atlanticae*) and temporary wet tamarisk thickets (*Tamaricion senegalensis*), occurring in infra-thermotropical and low mesotropical upper arid to dry bioclimates, occasionally in lower subhumid; when in arid bioclimates seems to be necessary a shallow temporary hydromorphism on soils.
Dichrostachyo platycarpae – Acacietum caboverdeanae Rivas-Martínez, Lousã, J.C. Costa & M.C. Duarte 2017	Climactic phanerophytic deciduous microwoodland savanna community, growing on andosols and leptosols, on Santiago Island. It occurs in upper infra and thermotropical, upper arid, semiarid, and lower dry bioclimate.
Echietum vulcanori Rivas-Martínez, Lousã, J.C. Costa & M.C. Duarte 2017	Shrub climactical open savanna community dominated and well characterized by the local endemic *Echium vulcanorum*.
Echio hypertropici – Euphorbietum tuckeyanae Rivas-Martínez, Lousã, J.C. Costa & M.C. Duarte 2017	Edaphoxerophytic and climatical tall, crowded shrub savanna community of Serra da Malagueta (Santiago Island), growing on andosols and coluvisols, in tropical xeric and occasionally some years pluviseasonal, thermotropical, semiarid to dry euhyperoceanic bioclimate.

Echio stenosiphonis – Euphorbietum tuckeyanae Rivas-Martínez, Lousã, J.C. Costa & M.C. Duarte 2017	Climactical shrub savanna community, growing on leptic andosols near the summit of de São Vicente Island, in topographical tropical cloudy, thermotropical, upper arid, euhyperoceanic bioclimate.
Erysimo caboverdeanae – Periplocetum chevalieri Rivas-Martínez, Lousã, J.C. Costa & M.C. Duarte 2017	Climactical and edaphoxerophillous tall shrub savanna community, occurring on leptosols and leptic andosols in Fogo Island, in tropical xeric, thermo-mesotropical, semiarid to upper dry, euhyperoceanic bioclimate.
Euphorbio azoricae – Festucion petraeae Lüpnitz 1976	Vegetation of salt-sprayed coastal cliffs of the Azores. Termomesotemperate levels.
Euphorbion melliferae Capelo, J.C. Costa, Jardim, Sequeira, Aguiar & Lousã 2003	Microphyllous caulirosulate communities with woody habit and large leaves, proper interrupt the forest canopy.
Fico gnaphalocarpae – Acacion caboverdeanae Rivas-Martínez, Lousã, J.C. Costa & M.C. Duarte 2017	Deciduous open micro-woodlands savanna, climactical, edaphoxerophilous or seasonally hygrophilous with fleeting superficial temporal hydromorphy; growing in upper infra and thermotropical arid to dry bioclimates; developed on lithic or regosolic soils.
Forsskaoleo procridifoliae – Ficetum gnaphalocarpae Rivas-Martínez, Lousã, J.C. Costa & M.C. Duarte 2017	Micro-mesophanerophytic woodland savanna community, growing on seasonal temporary moist shallow soils, on plains and torrents with fleeting superficial temporary hydromorphy.
Globulario amygdalifoliae – Periplocion chevalieri Rivas-Martínez, Lousã, J.C. Costa & M.C. Duarte 2017	Desertic and xeric climactical tropical and edaphoxerophilous tall shrub savanna community; in thermo-mesotropical, occasionally lower supratropical, from upper arid to upper dry, locally some years low subhumid (Fogo Island); developed on leptosols, andosols, colluvial and tephric soils.
Heteropogonetum melanocarpi Rivas-Martínez, Lousã, J.C. Costa & M.C. Duarte 2017	Xeromorphic perennial short grassland savanna, occurring in thermo to lower mesotropical semiarid to dry bioclimates, in Santiago and Fogo Islands, growing on leptic andosols.
Holco rigidi – Brachypodietum gaditanae Aguiar & F. Prieto in F. Prieto, Aguiar & Dias 2012	Thermotemperate or thermomediterranean mesoxerophilous grassland of *Brachypodium gaditanum*, in less disturbed habitats co-dominated by *Holcus rigidus*. Its habitats are small platforms in rock outcrops with soil accumulations provided by solifluction and earth flows.
Hordeion leporini Br.-Bl. in Br.-Bl., Gajewski, Wraber & Walas 1936 corr. O. Bolòs 1962	Mediterranean ruderal winter-annual grasslands.
Hyparrhenion hirtae Br.-Bl., Rozeira & P.Silva 1956	Grassland rich in perennial tall grasses, deep soils of rocky/ clayey cliffs, abandoned fields with some nitrification in infra to mesomediterranean semiarid to subhumid bioclimate; Mediterranean distribution.
Isoplexido sceptri – Euphorbietum melliferae Capelo, J.C. Costa, Jardim, Sequeira, Aguiar & Lousã 2003	Microphyllous caulirosulate community, endemic from Madeira Island, on rocky basaltic walls, in mesotemperate to low supratemperate, humid to hiper-humid, in laurissilva clearances or places submitted to landslides.
Launaeo thalassicae – Euphorbietum tuckeyanae Rivas-Martínez, Lousã, J.C. Costa & M.C. Duarte 2017	Edaphoxerophilous closed shrub savanna community, on leptosols and basalt on rocks and near vertical walls exposed to moist and wet winds.
Loto latifolii – Artemisietum gogonei Rivas-Martínez, Lousã, J.C. Costa & M.C. Duarte 2017	Climactical closed shrub savanna community, growing on leptics andosols, in upper thermotropical and lower mesotropical, semiarid to lower dry, euhyperoceanic bioclimate in Santo Antão Island.
Melanoselino bischoffii – Globarietum amygdalifoliae Rivas-Martínez, Lousã, J.C. Costa & M.C. Duarte 2017	Edaphoxerophilous nanophanerophytic short savanna community, growing on leptosols and basalt walls exposed to moist and wet winds, in tropical pluviseasonal, thermotropical, dry, euhyperoceanic bioclimate, in Santo Antão Island.
Monizia edulis community	In vertical basaltic walls in the road for Curral das Freiras, alongside with other taxa either from moister and higher areas as well as from drier and lowest parts of the island.
Onorpodion castellani Br.-Bl. & O. Bolòs 1958 corr. Rivas-Martínez, T.E. Díaz, Fernández-González, Izco, Loidi, Lousã & Penas 2002	Ruderal indifferent edaphic thistles communities, in abandoned fields and crops on amended deep soils, with a short period of hydromorphy, in infra - supra -mediterranean bioclimate with Mediterranean West Iberian, Baetic and Maghrebi distribution.
Scrophulario sublyratae – Lavateretum arboreae J.C. Costa, Capelo, Neto, Arsénio & Lousã 2012	Ornitocoprophilous, nitrophilous and aerohaline association; islands reefs and coastal cliffs with rough sea, in the Sadensean-Portuguese Subprovince frontier; indifferent edaphic, in granites, syenites, and limestones in Mediterranean pluviseasonal -oceanic, euhyperoceanic, lower mesomediterranean to upper thermomediterranean, dry bioclimate.
Sinapidendro angustifolii – Aeonion glutinosi Capelo, J.C. Costa, Lousã, Fontinha, Jardim, Sequeira & Rivas-Martínez 2000	Madeiran chomophytic and chasmophytic succulent-rich scrub on volcanic rock substrates and walls.
Tetraeno waterlotii – Sarcostemmetum daltonii Rivas-Martínez, Lousã, J.C. Costa & M.C. Duarte 2017	Climactical and edafoxerophilous close dwarf shrub savanna community, occurring in Brava Island on leptosols in lower thermotropical arid, euhyperoceanic bioclimate.

Tolpido succulentae – Agrostion congestiflorae Aguiar & F. Prieto in F. Prieto, Aguiar & Dias 2012	Thermomediterranean, occasionally thermoremperate, perennial graminoid communities of cliffs and landslide scarps.
Vulpietalia Pignatti 1953	Mediterranean and Ibero-Atlantic ephemeral therophytic vegetation on coastal sand dunes under influence of salt spray.

Table S4: Taxa of the sampled *Daucinae* tribes and their sampling locations (A, African Territory; Eu, European Territory; Az, Azores; CV, Cabo Verde; EU, Lu, mainland Portugal; Ma, Madeira; D, Desertas; F, Fogo; Fa, Faial; Fl, Flores; MA, Madeira Island; Pi, Pico; S, Santiago; SA (Az), Santa Maria; SA (CV), Santo Antão; SN, São Nicolau; SV, São Vicente).

Taxa	Location
Daucus carota subsp. *azoricus*	EU, Lu, Az: Fa, Horta, Castelo Branco
	EU, Lu, Az: Fl, Santa Cruz das Flores
	EU, Lu, Az: Pi, Madalena, Areia Larga
	EU, Lu, Az: SA, Vila Do Porto, São Pedro
Daucus carota subsp. *carota*	EU, Lu, Mainland: Lisboa, Lisboa, Tapada da Ajuda
Daucus carota subsp. *gummifer*	EU, Lu, Mainland: Leiria, Alcobaça, Praia da Polvoeira
	EU, Lu, Mainland: Leiria, Nazaré, Praia do Norte
	EU, Lu, Mainland: Leiria, Peniche, Papôa
	EU, Lu, Mainland: Leiria, Pombal, Carriço
Daucus carota subsp. *halophilus*	EU, Lu, Mainland: Beja, Odemira, Almograve
	EU, Lu, Mainland: Beja, Odemira, Cabo Sardão
	EU, Lu, Mainland: Setúbal, Sines, Porto Covo
	EU, Lu, Mainland: Faro, Vila do Bispo, Cabo de São Vicente
	EU, Lu, Mainland: Lisboa, Cascais, Praia do Guincho
	EU, Lu, Mainland: Beja, Odemira, Praia do Carvalhal
Daucus carota subsp. *maximus*	EU, Lu, Mainland: Faro, Loulé, Monte da Charneca
	EU, Lu, Mainland: Faro, Loulé, Benafim Grande
	EU, Lu, Mainland: Faro, Vila do Bispo, Cabo de São Vicente
Daucus carota subsp. *sativus*	EU, Lu, Mainland: Lisboa, Lisboa, Tapada da Ajuda
Daucus crinitus	EU, Lu, Mainland: Setúbal, Setúbal, Serra da Arrábida, El Carmen
	EU, Lu, Mainland: Setúbal, Setúbal, Portinho da Arrábida
Daucus muricatus	EU, Lu, Mainland: Faro, São Brás de Alportel
	EU, Lu, Mainland: Lisboa, Lisboa, Tapada da Ajuda
Melanoselinum decipiens	EU, Lu, Ma: MA, Ponta de Sol, Paul Serra, Levada 25 Fontes
	EU, Lu, Ma: MA, Santana, Caldeirão Verde
	EU, Lu, Ma: MA, Santana, Pico Ruivo
	EU, Lu, Ma: MA, Porto Moniz, Ribeira da Janela
	EU, Lu, Ma: MA, São Vicente, Lameiros, Rota do Cal
	EU, Lu, Ma: MA, São Vicente
Monizia edulis	EU, Lu, Ma: D, Deserta Grande
	EU, Lu, Ma: MA, Câmara de Lobos, Curral das Freiras
	EU, Lu, Ma: MA, Câmara de Lobos, Cabo Girão
Pseudorlaya pumila	EU, Lu, Mainland: Faro, Vila do Bispo, Cabo de São Vicente
Tornabenea annua	A, CV: S
	A, CV: S, Tarrafal Serra da Malagueta
Tornabenea bischoffii	A, CV: SA, Paúl, Cova
	A, CV: SA, Ribeira Grande, Corda
	A, CV: SA, Ribeira Grande, Estraga
	A, CV: SA
Tornabenea insularis	A, CV: SN, Vila da Ribeira Brava, Monte da Sentinha
	A, CV: SN, Monte Gordo
	A, CV: SV, Monte Verde
Tornabenea ribeirensis	A, CV: SN, Tarrafal, Assumada de Mancebo
Tornabenea tenuissima	A, CV: F
